# Evaluation of a Pediatric Community-Acquired Pneumonia Antimicrobial Stewardship Intervention at an Academic Medical Center

**DOI:** 10.3390/antibiotics12040780

**Published:** 2023-04-19

**Authors:** Lauren Puzz, Emily A. Plauche, David A. Cretella, Virginia A. Harrison, Mary Joyce B. Wingler

**Affiliations:** 1 University of Mississipp School of Pharmacy, Jackson, MS 39216, USA; lpuzz@umc.edu; 2 Department of Pharmacy, University of Mississippi Medical Center, Jackson, MS 39216, USA; eplauche@umc.edu; 3 Department of Antimicrobial Stewardship, University of Mississipp Medical Center, Jackson, MS 39216, USA; dcretella@umc.edu; 4 Department of Pediatrics, School of Medicine, University of Mississippi Medical Center, Jackson, MS 39216, USA; vharrison@umc.edu

**Keywords:** community-acquired pneumonia, antibiotic stewardship, pediatric

## Abstract

(1) Background: Pneumonia is the leading diagnosis associated with antibiotic use in hospitalized children. The Infectious Diseases Society of America published pediatric community-acquired pneumonia (CAP) guidelines in 2011, but adherence to recommendations varies across institutions. The purpose of this study was to evaluate the impact of an antimicrobial stewardship intervention on antibiotic prescribing in pediatric patients admitted to an academic medical center. (2) Methods: This single-center pre/post-intervention evaluation included children admitted for CAP during three time periods (pre-intervention and post-intervention groups 1 and 2). The primary outcomes were changes in inpatient antibiotic selection and duration following the interventions. Secondary outcomes included discharge antibiotic regimens, length of stay, and 30-day readmission rates. (3) Results: A total of 540 patients were included in this study. Most patients were under five years of age (69%). Antibiotic selection significantly improved, with prescriptions for ceftriaxone decreasing (*p* < 0.001) and ampicillin increasing (*p* < 0.001) following the interventions. Antibiotic duration decreased from a median of ten days in the pre-intervention group and post-intervention group 1 to eight days in post-intervention group 2. (4) Conclusions: Our antibiotic stewardship intervention directed at pediatric CAP treatment resulted in improved antibiotic prescriptions and provides data that can be used to further educate providers at our institution.

## 1. Introduction

Community-acquired pneumonia (CAP) continues to be one of the most common infections and causes of hospitalization in the pediatric population. Although respiratory viruses are the most frequent pathogens identified in children with CAP, pneumonia is the leading diagnosis associated with antibiotic use in pediatric hospitals in the United States [1].

The most common bacterial pathogen to cause CAP in children is *Streptococcus pneumoniae.* Accordingly, the 2011 Infectious Diseases Society of America (IDSA) pediatric CAP guidelines recommend narrow-spectrum penicillins, such as ampicillin or penicillin G, for fully immunized patients and third-generation cephalosporins for infants and children who are not fully immunized or where local susceptibilities show a high level of penicillin resistance. The guidelines support the transition to oral step-down therapy and suggest a total antibiotic treatment duration of ten days [2]. However, multiple randomized control trials have since been published, supporting shorter durations of approximately five days for children with CAP [3,4,5,6].

Despite these guideline recommendations and evidence to support short antibiotic courses, many institutions have reported overuse of broad-spectrum antibiotics and prolonged durations associated with pediatric CAP treatment [7,8]. Cohen and colleagues saw decreased broad-spectrum antibiotic use and increased adherence to pediatric CAP guideline recommendations following an antimicrobial stewardship intervention [9].

In 2018, our institution designed and implemented local pediatric CAP treatment guidelines, and antimicrobial stewardship pharmacists began handshake stewardship with providers to aid with implementation. Handshake stewardship included performing prospective audits with feedback and rounding in person with providers when possible. The aim of this study was to evaluate the effect of antimicrobial stewardship interventions targeted at pediatric CAP on antibiotic prescribing, as well as to compare clinical outcomes before and after the implementation of these interventions.

## 2. Results

A total of 1000 patients were reviewed for inclusion in this study. Of these, 540 patients met the criteria (211 in the pre-intervention group, 101 in the post-intervention group 1, and 228 in the post-intervention group 2). The median age of patients was similar between groups (*p* = 0.858), and the majority of patients were under five years of age (69%) (Table 1). Blood culture obtainment increased from the pre-intervention group to post-intervention groups 1 and 2 (45% vs. 64% vs. 61%, *p* < 0.001). Respiratory cultures were infrequently obtained for all groups. In the two post-intervention groups, immunization status was assessed. In both groups, 92% of patients had received at least two doses of PCV and Hib vaccinations (*p* = 1.000).

Ceftriaxone was the most common antibiotic prescribed in all groups but was significantly lower in both post-intervention groups (*p* < 0.001) (Table 2). In the pre-intervention group, combination therapy and azithromycin monotherapy were the next most common antibiotic regimens prescribed, and these also significantly decreased post-intervention. In contrast, ampicillin prescriptions significantly increased from <1% in the pre-intervention group to 23% and 32% in post-intervention groups 1 and 2, respectively (*p* < 0.001). Furthermore, in the post-intervention groups, antibiotics were more frequently de-escalated from broader-spectrum antibiotics such as ceftriaxone to ampicillin or amoxicillin (3% vs. 24% vs. 22%, *p* < 0.001). Similarly, discharge antibiotic prescriptions shifted significantly from third-generation cephalosporins to narrow-spectrum penicillins post-intervention. In the pre-intervention group, the most common discharge antibiotic was cefdinir (47%), which decreased to 9% and 5% in the post-intervention groups (*p* < 0.001). Amoxicillin prescriptions increased from 13% in the pre-intervention group to 48% and 46% in the post-intervention groups (*p* < 0.001).

The duration of therapy was similar between the pre-intervention group and post-intervention group 1 (median 10 vs. 10 days) but was significantly lower in post-intervention group 2 (median 8 days, *p* < 0.00001) (Table 2). The length of stay was significantly lower in the post-intervention groups (*p* = 0.005), and 30-day readmission was low across the groups (*p* = 0.076).

## 3. Discussion

This study evaluated the impact of an antimicrobial stewardship intervention on antibiotic selection and duration for the treatment of pediatric CAP at an academic pediatric hospital. The initiative, which consisted of the development of institutional treatment guidelines and implementing handshake stewardship with pediatric providers, proved to be beneficial in improving antibiotic use for children hospitalized with CAP. This was evidenced by a decrease in antibiotic duration and an improvement in antibiotic selection in both post-intervention groups. In addition to improvements in antibiotic prescribing, the length of stay was significantly lower in both post-intervention groups and 30-day readmission was less than 10% in all groups. However, based on these results, there is still room for improvement in antibiotic selection, duration of therapy, and microbiological work-up performed.

Our results demonstrated a significant increase in the use of ampicillin for children with CAP. This was sustained in both post-intervention groups. As described previously, the 2011 IDSA pediatric CAP guidelines recommend narrow-spectrum agents, such as ampicillin or penicillin G, for children who are fully immunized, primarily targeting *S. pneumoniae* [2]. Children who have received the pneumococcal conjugate vaccine (PCV) are protected against not only invasive pneumococcal disease, but also against penicillin-resistant serotypes such as serotype 19A [10,11]. Since the introduction of PCV options, penicillin resistance has significantly declined [10], and 97% of *S. pneumoniae* isolates are susceptible to penicillin based on the most recent antibiogram at our institution. Additionally, local vaccination rates are high, with 92% of patients in the two post-implementation groups having received at least two doses of a PCV and *Haemophilus influenzae* type b (Hib) vaccine at the time of admission for CAP. There are also clinical data supporting the use of narrow-spectrum therapy [12]. Williams and colleagues performed a large, retrospective cohort study of over 15,000 patients admitted with CAP to 43 children’s hospitals across the United States from 2005 to 2011 [12]. They compared narrow-spectrum therapy (ampicillin or penicillin G) to broad-spectrum therapy (ceftriaxone or cefotaxime). While the majority of patients (89.7%) received broad-spectrum antibiotics, no difference was found in clinical outcomes between those who received broad- or narrow-spectrum agents. The primary outcome evaluated the length of stay, which was three days in both groups (*p* = 0.11). Other outcomes assessed were admission to the intensive care unit (ICU) (1.1% vs. 0.8%, *p* = 0.26) and 14-day hospital readmission rates (2.3% vs. 2.4%, *p* = 0.76), which were similar in the broad-spectrum and narrow-spectrum groups, respectively.

Though our results demonstrated a significant increase in the use of narrow-spectrum penicillins for the treatment of CAP, the majority of patients received a third-generation cephalosporin across all groups. This is similar to what other studies have found regarding antibiotic selection [13]. A study conducted around the release of the 2011 IDSA pediatric CAP guidelines compared antibiotic selection 20 months before and 9 months after guideline publication [13]. A total of 2121 children were included. Third-generation cephalosporin use declined from 52.8% to 44.8% and penicillin/ampicillin use increased from 2.7% to 15.2%. While antibiotic prescribing was more aligned with the guidelines in the post-guideline time period, rates of third-generation cephalosporin prescribing remained high. It is appropriate to use these broad-spectrum agents in certain situations, such as for patients who are not up to date on their immunizations, those with a life-threatening infection, or those at institutions where penicillin resistance rates are high (>25%) [2]. However, based on our local vaccination and resistance rates, ampicillin should have been an appropriate initial antibiotic for most patients. Although not all fully immunized patients were empirically started on ampicillin, significantly more were de-escalated to ampicillin or amoxicillin following the implementation of the intervention. Despite this difference, the rate of de-escalation in the post-intervention groups was still lower than desired, with only 22–24% of patients being switched to narrower-spectrum agents. This is likely due to the absence of handshake stewardship in the pediatric hospital during the COVID-19 pandemic, which represents almost the whole second post-intervention group. Third-generation cephalosporins were not only the most common inpatient antibiotic used; they were also the most frequently prescribed discharge antibiotic in the pre-intervention group (cefdinir, 47%). In the post-intervention groups, <10% of patients received cefdinir. This coincided with amoxicillin prescriptions significantly increasing from 13% to 46–47% in the post-intervention groups, making discharge antibiotic selection one of the most successful aspects of the intervention.

Azithromycin prescribing also significantly decreased from the pre-intervention group to the post-intervention groups for monotherapy and combination therapy. Specifically focusing on monotherapy, there was a significant decline from 12% to 4–6%, which is low overall; however, monotherapy is not recommended with this agent due to the high rates of *S. pneumoniae* resistance (30% susceptibility based on the most recent institutional antibiogram). Azithromycin does provide adequate coverage for atypical pathogens, and our institutional guidelines state that it can be considered for patients with clinical or radiographic suspicion of atypical CAP, which is in line with the IDSA guidelines [2]. However, the addition of macrolide therapy to beta-lactam monotherapy does not appear to impact clinical outcomes for most patients [14]. Williams and colleagues compared the effectiveness of beta-lactam monotherapy to macrolide combination therapy in children hospitalized with CAP [14]. Of the 1418 patients included, 71.9% received beta-lactam monotherapy and 28.1% received macrolide combination therapy. There was no difference in the length of stay between groups (55 h vs. 59 h; unadjusted hazard ratio (HR) 1.01; 95% CI 0.90–1.14). This remained true even among planned subgroup analyses of patients at increased risk of atypical pneumonia (children older than five years, children with atypical bacteria detected, children admitted to the ICU, and children with acute wheezing). Additionally, almost 40% of children with an atypical organism detected did not receive a macrolide, highlighting the debatable need for this coverage in hospitalized children with CAP. The recommendation to withhold atypical coverage contrasts with common adult CAP antibiotic recommendations and may contribute to overprescribing of azithromycin by providers who practice in both adult and pediatric settings. While azithromycin use is low, continuing to discourage prescribing this agent for most children with CAP is reasonable based on resistance rates and its questionable impact on clinical outcomes.

The median duration of therapy decreased slightly from ten days in the pre-intervention and first post-intervention groups to eight days in the second post-intervention group. It is not uncommon for children with CAP to receive antibiotics for ten days or longer [15]. Shapiro and colleagues performed a retrospective evaluation using a claims database of publicly insured patients in 11 states. Their results showed that 82.8% of outpatient antibiotic prescriptions for children with CAP were written for a duration of 10 days, whereas only 10.5% were written for a shorter duration [15]. However, multiple randomized control trials supporting shorter durations have since been published [3,4,5,6]. Pernica and colleagues published the SAFER trial in 2021, which compared five- and ten-day courses of high-dose amoxicillin for pediatric patients diagnosed with CAP in the emergency department (ED) [3]. The primary outcome of a clinical cure at 14 to 21 days occurred in 84.1% of patients treated for five days and 85.7% of patients treated for ten days (risk difference, 0.023; 97.5% CL, −0.061). Another study published in 2021, the CAP-IT trial, was performed to determine whether lower-dose amoxicillin (35–50 mg/kg/day) was non-inferior to high-dose amoxicillin (70–90 mg/kg/day) and whether three days of treatment was non-inferior to seven days of treatment for children with CAP discharged from the ED or inpatient ward [4]. Clinically indicated antibiotic re-treatment for respiratory infection occurred in 12.5% of patients in both duration groups (difference, 0.1% (one-sided 95% CI –∞ to 3.9)), showing three days of therapy for children with CAP was non-inferior to seven days. There was no significant difference in outcomes between the amoxicillin dose (*p* = 0.46) or duration groups (*p* = 0.59). Similar to the SAFER trial, the SCOUT-CAP trial compared short (five-day) and standard (ten-day) antibiotic courses for the treatment of CAP in children in an outpatient clinic, urgent care, or ED setting [5]. Patients in the short-course group had a higher probability of a more desirable outcome [69% (95% CI, 63–75)] and a lower median number of antibiotic resistance genes compared to patients in the standard-course group (*p* = 0.01). The authors concluded that five days of antibiotic therapy was superior to ten days for children being treated for CAP in an outpatient setting.

The above studies provide data supporting short antibiotic durations for pediatric CAP treatment, but their application for hospitalized children with CAP may be limited since they were primarily completed in an outpatient setting. Another randomized control trial performed by McCallum and colleagues aimed to determine whether an extended antibiotic course was superior to a standard course of antibiotics for children hospitalized with uncomplicated CAP [6]. Patients received one to three days of intravenous (IV) antibiotics followed by oral amoxicillin/clavulanate (80 mg/kg/day amoxicillin) for a total of 13 to 14 days (extended group) or 5 to 6 days (standard group). Clinical cure occurred in 77.9% and 81.3% of patients in the extended and standard groups, respectively (relative risk, 0.96; CI, 0.86–1.07). In this trial, extended courses of antibiotics provided no additional advantage in achieving a clinical cure for children hospitalized with CAP. Although IDSA guidelines empirically recommend parenteral antibiotics for hospitalized children with CAP, this study provides evidence in favor of quickly transitioning these patients to oral antibiotics [2,6]. Approximately 83% of patients in both groups received IV antibiotics for two days or fewer before transitioning to oral amoxicillin/clavulanate to complete treatment. In a retrospective cohort study evaluating hospitalized children with CAP, Same and colleagues compared treatment failure between short-course (median six days) and prolonged-course (median ten days) antibiotics [16]. In the short-course group, 3% experienced treatment failure and 2% were readmitted due to CAP, whereas 6% experienced treatment failure and 3% were readmitted in the prolonged-course group. No difference in treatment failure was observed between the two groups. Based on the results of these studies focused on CAP treatment for hospitalized children, recommending shorter antibiotic durations for patients who show initial improvement is appropriate. Because the current median duration of therapy was eight days in post-intervention group 2, future interventions, including education and prospective audit with feedback, will continue to target durations of therapy.

The use of diagnostic cultures was identified as an area for improvement. The rate of blood culture obtainment increased from the pre-intervention group (45%) to the first and second post-intervention groups (64% and 61%, respectively). Our institutional guidelines suggest only obtaining blood cultures for patients with complicated pneumonia or those requiring admission to the ICU, as these patients are at an increased risk of having positive blood cultures compared to patients with uncomplicated CAP [1]. Since both of these groups were excluded from this study, the number of blood cultures performed should have been close to zero. Only about 2–7% of children with CAP will have positive blood cultures [1]. Therefore, the likelihood of patients included in this study having true bacteremia is low, and obtaining blood cultures for these patients leads to unnecessary use of hospital resources and costs. In addition, blood cultures that are positive with common contaminants such as coagulase-negative staphylococcus may lead to confusion when making treatment decisions. Similar to our study, Iroh Tam and colleagues evaluated adherence to pediatric CAP treatment guidelines [8]. For microbiological work-up, 61% of patients had blood cultures performed and only two were positive. One was positive for *S. pneumoniae*, which was considered to be a true positive, whereas the other was positive for a Micrococcus species and was determined to be a contaminant. This further supports reserving blood cultures for patients with complicated CAP or severe illness. Based on the increase in blood culture obtainment in the post-intervention groups of our study, additional education is needed. This education should be given to the emergency department and internal medicine providers, since they are most involved in the admission process for children with CAP. The rate of other diagnostics, such as biomarkers and polymerase chain reaction (PCR) panels, was not assessed, though institutional guidelines recommend against their use.

Overall, our study demonstrated that antimicrobial stewardship interventions can positively impact the management of CAP in hospitalized children, adding to the body of evidence supporting these interventions [9,17]. Cohen and colleagues implemented an antibiotic stewardship initiative focused on pediatric CAP diagnosis and treatment at their pediatric community health center by providing a one-day seminar for primary care pediatricians [9]. Additionally, a summary of recommendations was provided to the participants of the seminar. Following the provider education, there was a significant decrease in azithromycin use (23% vs. 14%) and an increase in amoxicillin use (57% vs. 66%) [9]. At another institution, Rossin and colleagues created clinical pathways, or one-page treatment algorithms, for the pediatric ED and acute care units as an antimicrobial stewardship initiative to try to improve antibiotic prescribing for CAP [17]. Implementation of the clinical pathway led to significantly lower macrolide use (66.7% to 0%). There were also improvements in rates of third-generation cephalosporin (77.8% to 38.5%) and amoxicillin (33.3% to 61.5%) prescriptions, but the differences were not significant. At our institution, the results of this study will be used to support antimicrobial stewardship efforts going forward in the children’s hospital. In addition, further evaluations may be warranted in the future if vaccine hesitancy increases across the United States, as this would change the landscape of antibiotic selection for CAP.

Formal presentations focused on pediatric CAP treatment were given to providers prior to and following the implementation of the guidelines, which is a strength of our study. Continuing provider education seemed to be beneficial as improvements in antibiotic use were sustained across both post-intervention groups, even while handshake stewardship interventions were absent during the COVID-19 pandemic. The educational presentation performed during the second post-intervention group was primarily focused on the appropriate treatment duration of pediatric CAP, which may have been a factor in the decrease in the duration of therapy seen in the second intervention group. Repeat exposure to information over prolonged periods of time benefits memory, and therefore, follow-up education is an important tool that can be used to increase the likelihood of the adoption of new stewardship recommendations [18,19]. Educational programs are an important part of antimicrobial stewardship initiatives, and more time spent on an educational intervention along with follow-up education results in better outcomes [19]. By combining provider education with pharmacist intervention through prospective audits and feedback, our study was successful in changing prescribing practices for pediatric patients hospitalized with uncomplicated CAP.

However, this study does have several limitations. Due to the retrospective nature of the study, we had to rely on provider documentation for the diagnosis of CAP, and the number of readmissions could have been underestimated if patients were admitted to an outside facility. There was also an interruption in stewardship activities due to the COVID-19 pandemic, which made it difficult to assess how much of an impact handshake stewardship had and likely contributed to low de-escalation rates. Since immunization status is unknown in the pre-intervention group, we are less able to assess the appropriateness of antibiotic selection for those patients. Although the numbers of blood and respiratory cultures performed were collected, the results of these tests were not collected, so the rate of positive cultures and most common bacterial pathogens among children admitted with CAP at our institution is unknown. Information regarding the collection or results of respiratory viral panels was not obtained. This limits our knowledge of the number of patients who had confirmed viral illnesses.

## 4. Materials and Methods

### 4.1. Study Design

This study was a single-center, pre/post-intervention quality improvement project evaluating pediatric patients admitted to the University of Mississippi Medical Center (UMMC) who were diagnosed with CAP. Three time periods were evaluated. The pre-intervention group included patients admitted between 1 July 2015 and 30 June 2016. The first post-intervention group included patients admitted from 1 January 2017 to 30 September 2019, and the second post-intervention group included patients admitted from 1 January 2020 to 28 February 2022. We collected age, immunization status, hospital length of stay, whether blood and respiratory cultures were obtained, inpatient antibiotics selected, discharge antibiotics selected, duration of therapy, whether antibiotics were de-escalated during therapy, length of stay, and 30-day readmission.

### 4.2. Study Setting

UMMC houses the state’s only children’s hospital, Children’s of Mississippi. Children’s of Mississippi is a 250-bed academic medical facility consisting of two patient care towers and the state’s only pediatric ED.

### 4.3. Inclusion/Exclusion Criteria

Patients were eligible for inclusion if they were admitted to Children’s of Mississippi with a primary diagnosis of uncomplicated CAP. Patients were excluded if they met any of the following criteria: admission to the pediatric intensive care unit (PICU) during this period of hospitalization; diagnosed pleural effusion or empyema; active COVID-19 infection (post-intervention group 2 only); or if pneumonia was associated with cystic fibrosis, sickle cell disease, or febrile neutropenia or occurred post-influenza infection.

### 4.4. Interventions

Prior to 2018, there were no institutional guidelines for pediatric CAP in place at UMMC. Based on the 2011 IDSA pediatric CAP guidelines, a multidisciplinary team, including hospitalists, infectious diseases providers, and the antimicrobial stewardship program, developed guidelines that recommend ampicillin or ceftriaxone empirically based on the patient’s immunization history. Patients were considered to have up-to-date immunizations if at least two doses of PCV and Hib vaccines were documented. Alternative antibiotic agents were recommended for patients with severe penicillin allergies. Our institution-specific guidelines recommended a total antibiotic duration of five to seven days for patients who respond within the first 48–72 h of antibiotic initiation. Recommendations for microbiologic testing were also provided, which suggested obtaining blood cultures only in patients with complicated pneumonia or those requiring admission to the ICU, and respiratory cultures only in patients who could provide a sputum sample. These guidelines, along with handshake stewardship, were implemented in Children’s of Mississippi in 2018. CAP-targeted presentations were performed at the time of guideline publication in 2018 (pre-intervention), in January 2020 (at the beginning of post-intervention group 2), and in November 2020 (during post-intervention group 2). The pre-intervention education primarily focused on antibiotic selection, while the post-intervention educational presentations highlighted new data on antibiotic duration for patients with CAP. In addition, educational handouts for CAP were distributed during Antibiotic Awareness Week in 2019 and 2021.

### 4.5. Outcomes

The primary outcome was the effect of this antimicrobial stewardship intervention targeting pediatric CAP antibiotic prescribing on inpatient antibiotic selection and duration. Secondary outcomes included choice of discharge antibiotic agent, length of stay, and 30-day readmission rates.

### 4.6. Data Analysis

Categorical data were evaluated using a Chi-square test. Continuous data were evaluated using either an ANOVA or Kruskal–Wallis test, as appropriate. Statistical significance was defined as a *p*-value of <0.05.

## 5. Conclusions

This retrospective, pre/post-intervention study evaluated the impact of implementing institution-wide guidelines along with handshake stewardship on the treatment of children with CAP. As a result of this intervention, there were many improvements in antibiotic prescribing. The median duration of therapy also decreased but remains higher than recommended by our institutional guidelines. Based on the results of this study, our antimicrobial stewardship initiative was shown to be beneficial in optimizing the treatment of pediatric CAP and identified areas of improvement to target further provider education and intervention.

## Figures and Tables

**Table 1:** Baseline characteristics.

Variable	Pre-Intervention (n = 211)	Post-Intervention Group 1 (n = 101)	Post-Intervention Group 2 (n = 228)	*p*-Value
Age in years, mean (SD)	3.84 (0.26)	4.12 (0.39)	3.95 (0.29)	0.858
Blood culture performed, n (%)	95 (45)	65 (64)	139 (61)	<0.001
Respiratory culture performed, n (%)	0	0	7 (3)	0.008
Immunizations up to date, n (%)	-	93 (92)	209 (92)	1.000

SD = standard deviation.

**Table 2:** Treatment characteristics and clinical outcomes.

Variable	Pre-Intervention (n = 211)	Post-Intervention Group 1 (n = 101)	Post-Intervention Group 2 (n = 228)	*p*-Value
Inpatient antibiotics, n (%)				
Ampicillin	2 (<1)	23 (23)	73 (32)	<0.001
Amoxicillin	1 (<1)	10 (10)	11	0.003
Ceftriaxone	130 (62)	39 (39)	98 (43)	<0.001
Cefdinir	2	1 (1)	-	-
Azithromycin	26 (12)	6 (6)	10 (4)	<0.001
Clindamycin	2	1 (1)	6	0.504
Combination therapy	47 (22)	21 (21)	24 (11)	0.003
None	1	-	2	-
Other	-	-	5	-
Discharge antibiotics, n (%)				
Amoxicillin	28 (13)	48 (48)	106 (46)	<0.001
Amoxicillin/clavulanate	8 (4)	18 (18)	47 (21)	<0.001
Cefdinir	99 (47)	9 (9)	11 (5)	<0.001
Azithromycin	32 (15)	11 (11)	14 (6)	0.008
Clindamycin	2 (<1)	3 (3)	10 (4)	0.09
Levofloxacin	-	1 (1)	4 (2)	-
Combination therapy	35 (17)	10 (10)	14 (6)	0.002
Other	-	-	4 (2)	-
None (completed inpatient)	7 (3)	-	25 (11)	-
Antibiotic de-escalation performed, n (%)	6 (3)	24 (24)	50 (22)	<0.001
Duration of antibiotic therapy, median (IQR)	10 (7–10)	10 (9–11)	8 (7–10)	<0.00001
Length of stay, mean (SD)	4.90 (0.87)	2.07 (0.26)	1.92 (0.09)	0.005
30-day readmission, n (%)	4 (2)	7 (7)	12 (5)	0.076

IQR = interquartile range; SD = standard deviation.

## Data Availability

Not applicable.
